# The significance of time interval between perioperative SOX/XELOX chemotherapy and clinical decision model in gastric cancer

**DOI:** 10.3389/fonc.2022.956706

**Published:** 2022-12-23

**Authors:** Jun-Bing Chen, Zi-Ning Liu, Yin-Kui Wang, Fei Shan, Shuang-Xi Li, Yong-Ning Jia, Kan Xue, Ru-Lin Miao, Zhe-Min Li, Zhou-Qiao Wu, Xiang-Ji Ying, Yan Zhang, Zi-Yu Li, Jia-Fu Ji

**Affiliations:** Key Laboratory of Carcinogenesis and Translational Research (Ministry of Education/Beijing), Gastrointestinal Cancer Center, Peking University Cancer Hospital and Institute, Beijing, China

**Keywords:** gastric cancer, neoadjuvant chemotherapy, adjuvant chemotherapy, perioperative period, nomograms, decision analysis

## Abstract

**Introduction:**

To investigate the influences of time interval between multimodality therapies on survival for locally advanced gastric cancer (LAGC) patients, 627 patients were included in a retrospective study, and 350 who received neoadjuvant chemotherapy (NACT) based on SOX (S-1 plus Oxaliplatin)/XELOX (Capecitabine plus Oxaliplatin) treatment, radical surgery, and adjuvant chemotherapy (AC) from 2005.01 to 2018.06 were eligible for analyses.

**Methods:**

Three factors were used to assess influences, including time interval from NACT accomplishment to AC initiation (PECTI), time to surgery after NACT accomplishment (TTS), and time to adjuvant chemotherapy after surgery (TAC).

**Results:**

Concerning PECTIs, 99 (28.29%) experienced it within 9 weeks, 188 (53.71%) within 9–13 weeks, 63 (18.00%) over 13 weeks. Patients’ 5-year overall survival (OS) significantly decreased as trichotomous PECTI increased (78.6% *vs* 66.7% *vs* 55.7%, P = .02). Analogously, there was a significant decrease for dichotomous TTS (within *vs* over 5 weeks) in OS (P = .03) and progression free survival (PFS) (P = .01) but not for dichotomous TAC (within *vs* over 6 weeks) in OS and PFS (P = .40). Through multivariate Cox analyses, patients with PECTI over 13 weeks had significantly worse OS (P = .03) and PFS (P = .02). Furthermore, extended TTS had significantly worse OS and PFS but insignificantly worse OS and PFS than extended TAC. Therefore, gastric patients receiving perioperative SOX/XELOX chemotherapy and surgery with extended PECTI over 9 weeks or TTS over 5 weeks would have a negative correlation with PFS and OS, and worse when PECTI over 13 weeks. Nomograms (including PECTI, ypT, ypN, Area Under Curve (AUC) = 0.81) could predict patient survival probability and guide intervention with net benefit.

**Discussion:**

In control of PECTI, TTS could be extended appropriately, and shortened TAC might make a remedy, and delayed TAC might be allowed when TTS was shortened.

## 1 Introduction

Gastric cancer is the fifth most common cancer with the fourth highest mortality worldwide according to Global Cancer Statistics (1). More than 50% of cases occur in East Asian countries, and most of them suffer from advanced gastric cancer with high survival risks. Surgery alone cannot solve the problem, and multimodal therapies are essential. The current preferred care for locally advanced gastric cancer (LAGC) is perioperative chemotherapy plus surgery (2, 3), based on some clinical trials, such as MAGIC (4), FNCLCC (3), PRODIGY (5), and RESOLVE (6). Perioperative chemotherapy (PC) consists of neoadjuvant chemotherapy (NACT) and adjuvant chemotherapy (AC).

**Figure 1:**
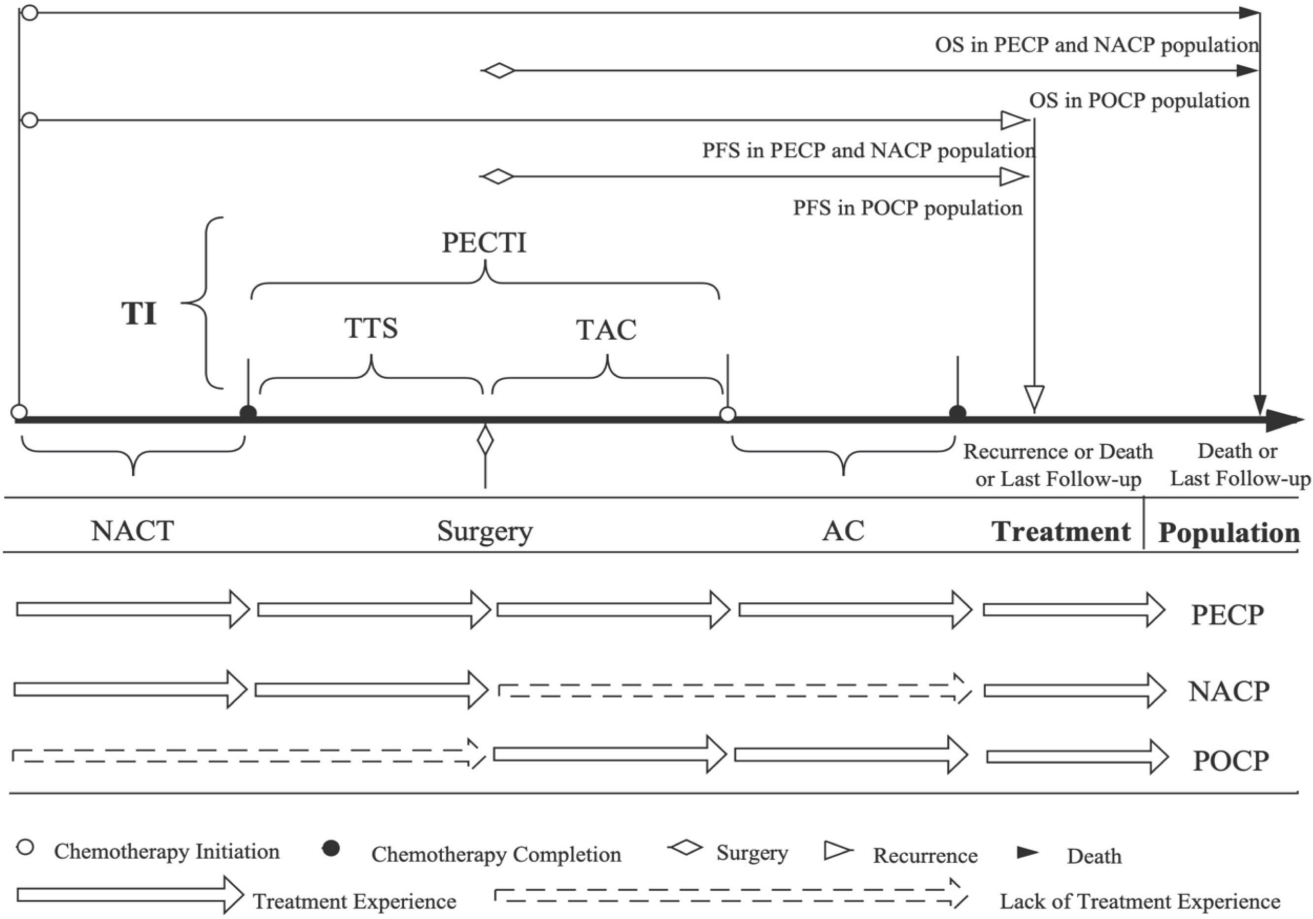
The definition and illustration of chemotherapy-related time and population. The concept of ‘TI’ contains PECTI, TTS, TAC, and TTS plus TAC is equal to PECTI. Treatment consists of NACT, surgery, and AC. ‘Population’ contains PECP, NACP, and POCP. In the PECP and NACP populations, PFS is the period from the initiation of NACT to the recurrence, metastasis, death, or the last follow-up, and OS is to the death or the last follow-up. In the POCP population, PFS is the time span from surgery to recurrence, and OS is to death or the last follow-up. OS, overall survival; PFS, progression-free survival; TI, time interval; PECTI, perioperative chemotherapy time interval; TTS, time to surgery; TAC, time to adjuvant chemotherapy; NACT, neoadjuvant chemotherapy; AC, adjuvant chemotherapy; PECP, perioperative chemotherapy population; NACP, neoadjuvant chemotherapy population; POCP, postoperative chemotherapy population.

How to schedule time intervals among multimodal therapies is always a topic of focus for medical practitioners and patients, which tends to depend on subjective experience. Taking gastric cancer as an example, the time to surgery (TTS) after NACT is usually arranged for 3–6 weeks, and the time to AC (TAC) after surgery is arranged for 4–12 weeks, which is based on the initial clinical trial protocols mentioned above (4). TTS and TAC were separately defined as the time intervals from completion of NACT to initiation of surgery and from completion of surgery to initiation of AC. Then, the Perioperative Chemotherapy Time Interval (PECTI), consisting of TTS and AC, refers to the period from completion of NACT to initiation of AC (**Figure 1**).

Nevertheless, the clinical value of TTS and TAC in patients is in dispute, and whether PECTI is meaningful for survival is even more complicated. Concerning gastric cancer, there are few related outcomes worldwide. One study reported that TTS has no correlation with survival, although it presents higher odds of pathological complete response (pCR) when TTS is over 6 weeks (7). Another study reported that TAC did not significantly influence overall survival (OS) among patients over 8 weeks, 6–8 weeks, and within 6 weeks (8). Previously, our cancer center had already confirmed the optimal TTS within 3–5 weeks, neither too early nor too late, with a survival benefit but noneffectiveness at pCR for patients who composed the perioperative chemotherapy population (PECP, who received NACT, surgery, and AC) and the neoadjuvant chemotherapy population (NACP, who only received NACT and surgery) (9). However, while insignificant in the PECP population, TAC just had an increasing trend in the hazard ratio (HR) in the postoperative population (POCP, who only received surgery and AC) (10). Thus, this study will further investigate the clinical value of PECTI in OS and PFS, provide a comprehensive elucidation, and then develop a prognostic nomogram model to provide references for clinical decision-making.

## 2 Material and methods

### 2.1 Study population

**Figure 2:**
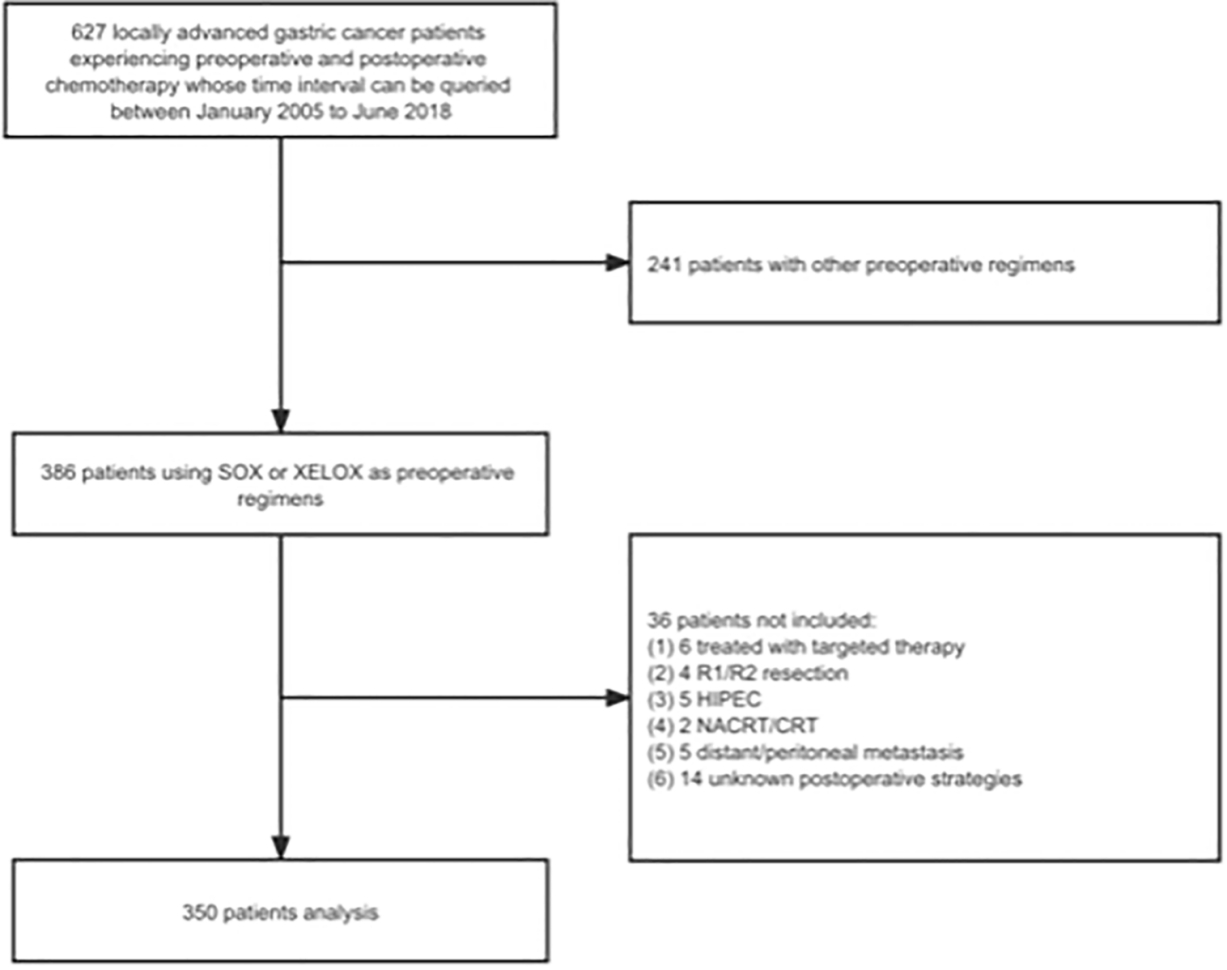
The study flow chart. SOX, S-1 plus oxaliplatin; XELOX, capecitabine plus oxaliplatin; HIPEC, hyperthermic intraperitoneal chemotherapy; NACRT, neoadjuvant chemoradiation therapy; CRT, chemoradiation therapy.

Our team selected 627 patients diagnosed with LAGC from January 2005 to June 2018. This was done at the gastrointestinal cancer center of the Peking University Cancer Hospital and Institute, and approval was granted by the Peking University Cancer Hospital Ethics Committee. The inclusion criteria were: (1) the patients diagnosed with non-metastatic LAGC by endoscopic biopsy prior to surgery and ulteriorly confirmed by postoperative pathology; (2) patients underwent preoperative and postoperative chemotherapy, curative gastrectomy surgery, all of which performed at our center; and (3) patients with complete recording of clinical and pathological data by professional stewards. The exclusion criteria were: (1) LAGC patients receiving preoperative chemotherapy other than S-1 plus oxaliplatin (SOX) or capecitabine plus oxaliplatin (XELOX) regimens; (2) patients experiencing R1/R2 resection or D0/D1/D1+ lymphadenectomy; (3) patients experiencing comprehensive treatment mixed with radiotherapy or immunotherapy or targeted therapy or hyperthermic intraperitoneal chemotherapy (HIPEC); (4) patients with suspected or confirmed metastasis before AC initiation; and (5) patients who experienced delayed surgery over 12 weeks after completion of NACT. Finally, 350 patients were eligible for deep analyses according to the criteria mentioned above (**Figure 2**). All patients were provided informed consent forms to allow the use and publication of medical data during their hospitalization. This observational study adhered to the tenets of the Declaration of Helsinki (11) and followed reporting guidelines (12).

### 2.2 Patients’ records

The patients’ multimodal therapies were standardized and recorded. After the diagnosis of LAGC through endoscopic biopsy and computed tomography (CT) or plus further laparoscopic examination, the treatment options and details of chemotherapy, surgery or other individualized approaches need the approval of specialists at multidisciplinary conferences in our center. The standard perioperative chemotherapy regimens were SOX and XELOX with one to six cycles of 3 weeks. If patients suffer from serious adverse effects or tumor progression, NACT may be interrupted and switched to evaluation of efficacy and surgery. Routine evaluation is also performed every two cycles. After the completion of NACT, curative total or subtotal gastrectomy plus D2 lymphadenectomy will be considered. The methods of anastomosis reconstruction include Roux-en-Y usually following total gastrectomy and Billroth-II usually following distal gastrectomy and others such as Billroth-I, jejunal interposition reconstruction. All surgical procedures were performed by two chief surgeons (J-FJ and Z-YL) from the same qualified and efficient surgery group. The continuous follow-up procedure *via* telephone quarterly was performed and managed by a dedicated team. If failing to contact the patients or their guardians in three attempts, they would make the consideration of loss of follow-up.

The extracted patients’ clinical and pathological information included patients’ preoperative basic medical data, perioperative time data, surgery details and complication data, postoperative pathology and survival outcomes. Preoperative basic medical data include gender, age, height and weight and calculated Body Mass Index (BMI), Eastern Cooperative Oncology Group performance status (ECOG PS), American Society of Anesthesiologists score (ASA), NACT cycle and regimens. Perioperative time-related data include PECTI, TTS, TAC and AC cycle. Surgery detail includes surgical approaches (laparoscopic and open surgery), resection range (total, distal, proximal, or abdominothoracic gastrectomy), lymph node dissected amount, operative duration, and blood loss. Complications recorded include infection and fever, hemorrhage, ascites, ileus, pleural effusion, pneumonia, lymphatic leakage, thrombosis, delirium, and condition of intensive care. Post-operable tumor pathology data include location (upper, middle, lower, and diffuse neoplasm), diameter (longest), differentiation grade (well, moderate, and poor or signet-ring), vascular involvement, postoperative pathological (yp), T and N stage. The Clavien-Dindo classification system would be used to classify these complications. Survival outcomes data include OS, PFS. PFS is the period from initiation of NACT to recurrence, metastasis, death or the last follow-up, and OS is the period from death or the last follow-up. In contrast, in the POCP population, PFS was the time span from surgery to recurrence, and OS was the time span from death or the last follow-up (**Figure 1**).

### 2.3 Statistical analyses

Descriptive statistics were performed for demographic data, including medians with interquartile ranges (IQRs) and percentages. The continuous variable cutoff of PECTI was decided considering both X-tile analyses based on Kaplan–Meier methods and clinical practice, taking practicability into consideration. The Shapiro–Wilk test was used for normality tests, and the Levene test was used for homogeneity tests of variance. The Kruskal–Wallis test for continuous variables obeying a nonnormal distribution and Pearson’s chi-square test or Fisher’s exact test for categorical variables were performed for comparison. The *post hoc* test of Nemenyi based on mean rank sums was used to perform multiple comparisons with equal variance assumed, and Tamhane’s T2 test was used with unequal variance assumed. Partitions of Pearson’s chi-square statistic were used for pairwise comparisons in the cross table. Kaplan–Meier curves and log-rank tests were performed for analyses of OS and PFS. The Cox proportional hazards regression model with a stepwise procedure was used to assess the effects of the prognostic factors by presenting hazard ratios (HRs) with 95% confidence intervals (CIs), and the significant factors (P <.05) in univariate analyses were further included in multivariate analyses. The subgroup forest plot based on survival rates was drawn to analyze PECTI (dichotomous, within or over 13 weeks) influences on survival. A prognostic nomogram model was constructed to generate survival probability at 1, 3, and 5 years. Among the Cox model, least absolute shrinkage and selection operator (Lasso) model and best subgroup regression (BSR) model, the combination of variables with maximum AUC (area under curve) and minimum backward stepwise Akaike information criterion (AIC) was chosen for the development of the prognostic nomogram model. The AUC in receiver operating curves (ROC) and Harrell’s concordance index (C-index) were used to estimate the model’s discrimination. Calibration plots were drawn to present the model’s estimation ability of outcome probabilities. Decision curve analyses (DCAs) and clinical impact curves (CICs) were used to present the net benefit of intervention based on the developed model prediction (13). N ∗ K cross-validation for the nomogram model was used. All tests were two-sided, and P <.05 was set as the significance level. All statistical analyses and plots were performed using R software (Version 4.1.1, R Foundation for Statistical Computing) (14), and X-tile software (version 3.6.1, Rimm Lab, Yale School of Medicine) was used to seek cutoff points (15).

## 3 Results

### 3.1 Patients’ characteristics

Among 350 eligible patients, the median age was 61 (IQR, 53 to 64), the median BMI was 23.74 (IQR, 21.63 to 26.00) kg/m^2^, and the median stay time at the hospital was 10 (IQR, 9 to 13) days. 273 (78%) of 350 patients were male, and 169 (48.29%) experienced more than two cycles of NACT. After surgery, 167 patients (47.71%) were disclosed with poor differentiation, and 58 (16.57%) with signet-ring, and 114 (32.57%) with vascular invasion, 27 (7.71%) with ypT0, 161 (46%) with ypN0, 24 (6.86%) with pCR, and 154 (44%) experienced more than four cycles of AC. All patients experienced at least one cycle of NACT (median, two cycles; range, one to six cycles) and AC (median, four cycles; range, one to 12 cycles).

The distribution in different TI groups was shown (**Figure S1**), and based on X-tile and clinical practice, PECTI was divided into trichotomous group (within 9 weeks, 9–13 weeks, and over 13 weeks), TTS dichotomous group (within 5 weeks and over 5 weeks), and TAC dichotomous group (within 6 weeks, over 6 weeks). The detailed demography and clinicopathologic characteristics are shown in **Table 1**, **Table S1**.

**Table 1:** Patient demography and clinicopathologic characteristics in the PECTI group.

Characteristics^a^		All patients	PECTI, weeks			
			≤9 weeks	9–13 weeks	>13 weeks	p
Patients Number		350	99/350 (28.29)	188/350 (53.71)	63/350 (18.00)	
Age, median [IQR], y		60.00 [53.00 to 64.00]	59.00 [53.00 to 64.00]	59.00 [52.00 to 64.00]	63.00 [57.50 to 67.00]	.004
BMI, median [IQR], (kg/m^2^)		23.74 [21.63 to 26.00]	24.30 [21.88 to 26.30]	23.43 [21.41 to 25.74]	23.94 [21.71 to 25.95]	.22
Operative Time [IQR], min		202.50 [171.00 to 243.75]	208.00 [165.00 to 245.00]	201.00 [173.50 to 244.25]	204.00 [174.50 to 236.00]	.98
Blood Loss [IQR], ml		100.00 [80.00 to 179.25]	100.00 [100.00 to 192.50]	100.00 [81.50 to 168.75]	100.00 [50.00 to 151.50]	.91
Stay-time [IQR], days		10.00 [9.00 to 13.00]	10.00 [9.00 to 12.00]	10.00 [9.00 to 13.00]	12.00 [9.00 to 16.00]	.02
Sex (%)	Male	273/350 (78.00)	78/99 (78.79)	146/188 (77.66)	49/63 (77.78)	.98
	Female	77/350 (22.00)	21/99 (21.21)	42/188 (22.34)	14/63 (22.22)	
ASA (%)	1	23/350 (6.57)	8/99 (8.08)	14/188 (7.45)	1/63 (1.59)	.49
	2	286/350 (81.71)	80/99 (80.81)	153/188 (81.38)	53/63 (84.13)	
	3	41/350 (11.71)	11/99 (11.11)	21/188 (11.17)	9/63 (14.29)	
ECOG (%)	0	264/350 (75.43)	82/99 (82.83)	138/188 (73.40)	44/63 (69.84)	.11
	1–3	86/350 (24.57)	17/99 (17.17)	50/188 (26.60)	19/63 (30.16)	
Gastrectomy (%) ^b^	Total	143/350 (40.86)	39/99 (39.39)	80/188 (42.55)	24/63 (38.10)	0.50
	Distal	183/350 (52.29)	53/99 (53.54)	98/188 (52.13)	32/63 (50.79)	
	Proximal	19/350 (5.43)	7/99 (7.07)	7/188 (3.72)	5/63 (7.94)	
	A-T	5/350 (1.43)	0/99 (0.00)	3/188 (1.60)	2/63 (3.17)	
Surgery Approach (%)	Laparoscopic	163/350 (46.57)	43/99 (43.43)	89/188 (47.34)	31/63 (49.21)	.74
	Open	187/350 (53.43)	56/99 (56.57)	99/188 (52.66)	32/63 (50.79)	
Complications, CD (%)	0	244/350 (69.71)	72/99 (72.73)	134/188 (71.28)	38/63 (60.32)	.06
	1–2	66/350 (18.86)	19/99 (19.19)	36/188 (19.15)	11/63 (17.46)	
	3–4	40/350 (11.43)	8/99 (8.08)	18/188 (9.57)	14/63 (22.22)	
Location (%)	Upper	105/350 (30.00)	32/99 (32.32)	52/188 (27.66)	21/63 (33.33)	.87
	Middle	54/350 (15.43)	12/99 (12.12)	32/188 (17.02)	10/63 (15.87)	
	Lower	177/350 (50.57)	52/99 (52.53)	96/188 (51.06)	29/63 (46.03)	
	Diffuse	14/350 (4.00)	3/99 (3.03)	8/188 (4.26)	3/63 (4.76)	
Diameter (%)	≤2 cm	159/350 (45.43)	37/99 (37.37)	92/188 (48.94)	30/63 (47.62)	.14
	2–5 cm	142/350 (40.57)	49/99 (49.49)	66/188 (35.11)	27/63 (42.86)	
	>5 cm	49/350 (14.00)	13/99 (13.13)	30/188 (15.96)	6/63 (9.52)	
Differentiation (%)	Well-Moderate	183/350 (52.29)	54/99 (54.55)	95/188 (50.53)	34/63 (53.97)	.78
	Poor	167/350 (47.71)	45/99 (45.45)	93/188 (49.47)	29/63 (46.03)	
Signet-ring (%)	No	292/350 (83.43)	87/99 (87.88)	150/188 (79.79)	55/63 (87.30)	.14
	Yes	58/350 (16.57)	12/99 (12.12)	38/188 (20.21)	8/63 (12.70)	
Vascular Invasion (%)	No	236/350 (67.43)	74/99 (74.75)	123/188 (65.43)	39/63 (61.90)	.16
	Yes	114/350 (32.57)	25/99 (25.25)	65/188 (34.57)	24/63 (38.10)	
ypT (%)	T0	27/350 (7.71)	8/99 (8.08)	15/188 (7.98)	4/63 (6.35)	.55
	T1	42/350 (12.00)	11/99 (11.11)	25/188 (13.30)	6/63 (9.52)	
	T2	64/350 (18.29)	21/99 (21.21)	28/188 (14.89)	15/63 (23.81)	
	T3	89/350 (25.43)	29/99 (29.29)	43/188 (22.87)	17/63 (26.98)	
	T4	128/350 (36.57)	30/99 (30.30)	77/188 (40.96)	21/63 (33.33)	
ypN (%)	N0	161/350 (46.00)	44/99 (44.44)	88/188 (46.81)	29/63 (46.03)	.05
	N1	70/350 (20.00)	29/99 (29.29)	34/188 (18.09)	7/63 (11.11)	
	N2	59/350 (16.86)	16/99 (16.16)	29/188 (15.43)	14/63 (22.22)	
	N3	60/350 (17.14)	10/99 (10.10)	37/188 (19.68)	13/63 (20.63)	
pCR (%)	No	326/350 (93.14)	92/99 (92.93)	175/188 (93.09)	59/63 (93.65)	.98
	Yes	24/350 (6.86)	7/99 (7.07)	13/188 (6.91)	4/63 (6.35)	
NACT Cycle (%)	≤2	181/350 (51.71)	51/99 (51.52)	98/188 (52.13)	32/63 (50.79)	.98
	>2	169/350 (48.29)	48/99 (48.48)	90/188 (47.87)	31/63 (49.21)	
NACT Regime (%)	SOX	238/350 (68.00)	74/99 (74.75)	123/188 (65.43)	41/63 (65.08)	.24
	XELOX	112/350 (32.00)	25/99 (25.25)	65/188 (34.57)	22/63 (34.92)	
AC Cycle (%)	≤4	196/350 (56.00)	50/99 (50.51)	108/188 (57.45)	38/63 (60.32)	.40
	>4	154/350 (44.00)	49/99 (49.49)	80/188 (42.55)	25/63 (39.68)	
AC Regime (%)	SOX or other ^c^	247/350 (70.57)	73/99 (73.74)	130/188 (69.15)	44/63 (69.84)	.71
	XELOX	103/350 (29.43)	26/99 (26.26)	58/188 (30.85)	19/63 (30.16)	

'aValues in parentheses and brackets are percentages and interquartile ranges, respectively. ^b^Calculated by Fisher exact test. ^C^Other regimens included S-1 (n = 13) and S-1 or capecitabine plus paclitaxel (n = 12).

PECTI, perioperative chemotherapy time interval; IQR, interquartile range; BMI, body mass index; ASA, American Society of Anesthesiologists; ECOG, Eastern Cooperative Oncology Group; AT, abdominal-thoracic surgery; CD, Clavein–Dindo Classification; pCR, pathological complete response; NACT, neoadjuvant chemotherapy; SOX, S-1 plus oxaliplatin chemotherapy; XELOX, capecitabine plus oxaliplatin chemotherapy; AC, adjuvant chemotherapy.

In *post hoc* test of Nemenyi, PECTI over 13 weeks was significantly different from group within 9 weeks (P = .013) and 9–13 weeks (P = .004). Concerning ‘Stay-time at hospital,’ Tamhane’s T2 test showed PECTI over 13 weeks was significantly different from PECTI within 9 weeks (P = .01) and 9–13 weeks (P = .02). In addition, borderline significant differences were shown for factors of ‘Complications’ (P = .06) and ‘ypN’ (P = .05) in PECTI group, but there was no apparent discrepancy between different PECTI subgroups by *post hoc* test of Chi-square partition method with adjusted α’ as a standard.

### 3.2 Survival analyses

**Figure 3:**
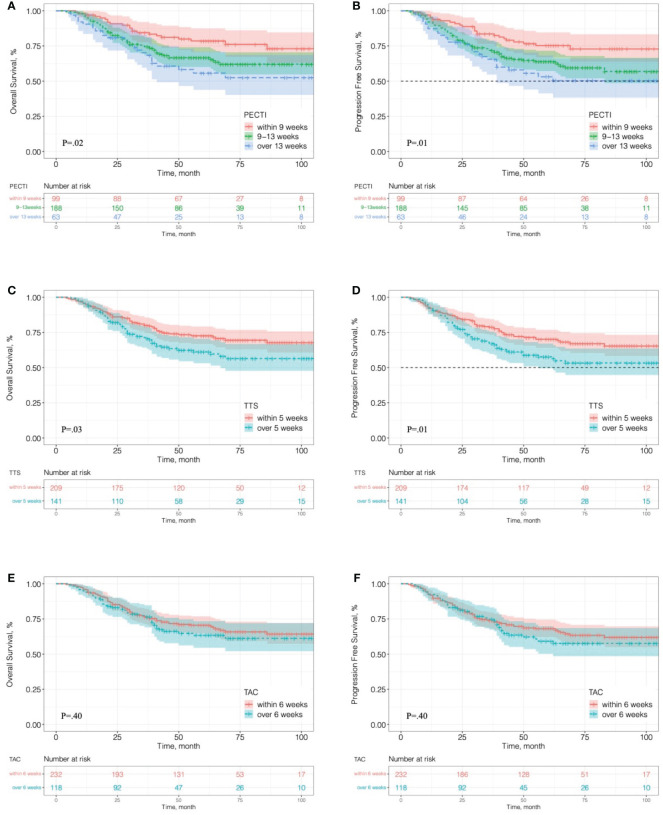
Kaplan–Meier analyses of overall survival and progression-free survival in different time interval groups. **(A)** OS in the trichotomous PECTI study group; **(B)** PFS in the trichotomous PECTI study group; **(C)** OS in the dichotomous TTS study group; **(D)** PFS in the dichotomous TTS study group; **(E)** OS in the dichotomous TAC study group; **(F)** PFS in the dichotomous TAC study group. PECTI, perioperative chemotherapy time interval; TTS, time to surgery; TAC, time to adjuvant chemotherapy.

**Figure 4:**
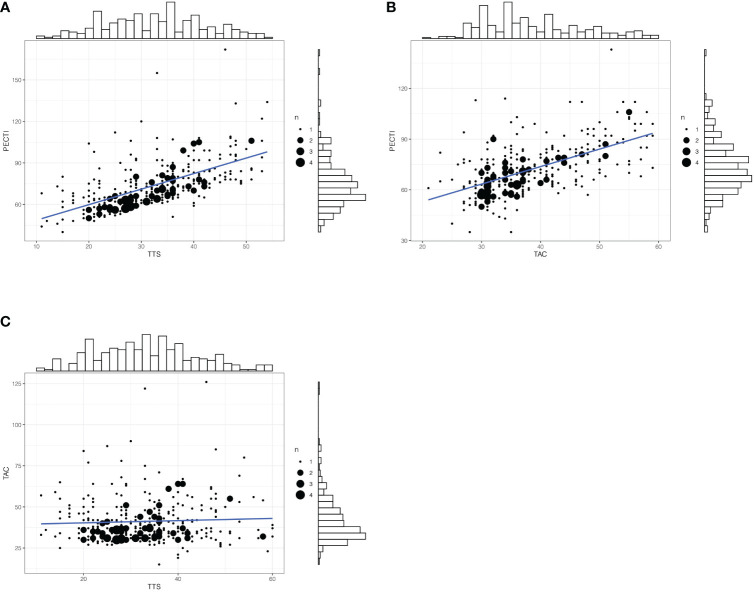
The matched counts plot with marginal histogram of time interval. **(A)** Correlationship between PECTI and TTS; **(B)** Correlationship between PECTI and TAC; **(C)** Correlationship between TAC and TTS. The plot would illustrate the collinearity between PECTI and TTS. The size of circles denotes the extent of overlapping points. PECTI, perioperative chemotherapy time interval; TTS, time to surgery; TAC, time to adjuvant chemotherapy.

In survival analyses, the median follow-up was 51 months (range, 4 to 128 months). Of 350 patients, 122 (34.8%) had suffered recurrence, and 111 (31.7%) died. Kaplan–Meier curves for OS and PFS are presented in **Figure 3**, which indicates that the patients’ 5 years OS was significantly different between PECTI within 9 weeks and 9–13 weeks and over 13 weeks (78.6% (95% CI, 70.6% to 87.4%) *vs* 66.7% (95% CI, 60.0 to 74.1%) *vs* 55.7% (95% CI, 43.8% to 70.9%), log-rank P = .02) (**Figure 3A**), and a similar result for PFS (**Figure 3B**). In addition, it was significantly different between TTS within and over 5 weeks in 5 years OS (72.7% (95% CI, 66.7% to 79.2%) *vs* 61.2% (95% CI, 53.1% to 70.5%), log-rank P = .03) (**Figure 3C**), and a similar result for PFS (**Figure 3D**). However, there was no significant difference between TAC within and over 6 weeks in 5 years OS (70.5% (95% CI, 64.7% to 76.9%) *vs* 63.3% (95% CI, 54.5% to 73.5%), log-rank P = .40) (**Figure 3E**), and similar for PFS (**Figure 3F**). In pairwise comparison, it was significantly different as PECTI over 13 weeks versus within 9 weeks for OS (P = .005) and PFS (P = .004), and likewise as PECTI with 9–13 weeks versus within 9 weeks for OS (P = .03) and PFS (P = .02). In addition, the dichotomous groups of PECTIs were also devised and analyzed, which showed that there was a significant difference in OS (P = .009) and PFS (P = .006) between PECTIs within and over 9 weeks (**Figures S2A, B**) and a significant difference in OS (P = .04) approaching a significant difference in PFS (P = .05) between PECTIs within and over 13 weeks (**Figures S2C, D**). Generally, patients with shorter PECTIs could have better PFS and OS and that extended PECTIs would decrease PFS and OS, similar to TTS. Nevertheless, shortened or extended TAC did not have significant influences on PFS and OS. Further analyses were performed to explore the relationship and causality among PECTI, TTS, and TAC (**Figure 4**). A highly linear relationship was found between PECTI and TTS.

Univariate and multivariate Cox proportional hazard models were devised to compare covariates’ influence on survival outcomes and are shown in **Table 2**. PECTI, TTS, TAC and 23 other clinically important covariates were included in the univariate Cox model through clinical-based selection. Extended PECTI had an increasing hazard and negative correlation with both OS and PFS. Compared to within 9 weeks, PECTI with 9–13 weeks induced a worse OS (HR = 1.71, 95% CI −1.05 to 2.78, P = .03) and PFS (HR = 1.75, 95% CI −1.10 to 2.78, P = .02), and PECTI over 13 weeks had a worse OS (HR = 2.26, 95% CI −1.27 to 3.99, P = .005) and PFS (HR = 2.22, 95% CI −1.29 to 3.84, P = .004). In addition, other covariates also had a significant influence (P <.05) on OS (11 covariates work) and PFS (10 covariates work) and were then included in the multivariate Cox model. In analyses of multivariate Cox, compared to within 9 weeks, PECTI over 13 weeks had a more negative correlation with OS (HR = 1.94, 95% CI −1.06 to 3.53, P = .03) and PFS (HR = 1.98, 95% CI −1.12 to 3.49, P = .02). Covariates such as ‘ypT’ and ‘ypN’ also had significant correlations with OS and PFS in the multivariate Cox model. In addition, an extra separate Cox analysis was performed between PECTI, TTS, and TAC and is shown in **Table S2**, which indicated that PECTI had borderline significant (P = .05) influences on both OS and PFS in the multivariate model.

**Table 2:** Univariate and Multivariate Survival Analyses Using Cox Proportional Hazards Model.

Characteristics	Progression Free Survival						Overall Survival					
	Univariate HR (95% CI)		P	Multivariate HR (95% CI)		P	Univariate HR (95% CI)		P	Multivariate HR (95% CI)		P
Age, years												
≤60	1.00						1.00					
>60	1.09	(0.76 to 1.55)	.65				1.15	(0.79 to 1.66)	.47			
BMI, kg/m^2^												
≤23.9	1.00						1.00					
>23.9	0.96	(0.68 to 1.38)	.84				0.86	(0.59 to 1.24)	.41			
Gender												
Male	1.00						1.00					
Female	1.15	(0.75 to 1.76)	.51				1.23	(0.80 to 1.90)	.34			
ASA Score												
1	1.00			1.00			1.00					
2	5.04	(1.24 to 20.44)	.02	3.51	(0.86 to 14.40)	.08	4.55	(1.12 to 18.45)	.03	3.04	(0.74 to 12.51)	.12
3	5.21	(1.20 to 22.57)	.03	3.41	(0.77 to 15.16)	.11	4.53	(1.04 to 19.81)	.05	2.63	(0.58 to 11.93)	.21
ECOG												
0	1.00			1.00			1.00					
1–3	1.59	(1.09 to 2.32)	.02	1.24	(0.83 to 1.86)	.29	1.48	(0.99 to 2.21)	.06			
Blood Loss, ml												
≤100	1.00			1.00			1.00					
>100	1.57	(1.10 to 2.24)	.01	1.10	(0.75 to 1.61)	.62	1.68	(1.15 to 2.43)	.007	1.20	(0.81 to 1.79)	.36
Operative Time, min												
≤200	1.00						1.00					
>200	1.37	(0.95 to 1.95)	.09				1.43	(0.98 to 2.09)	.06			
Postoperative Stay-time, days												
≤10	1.00						1.00					
>10	1.37	(0.96 to 1.96)	.08				1.54	(1.06 to 2.23)	.02	1.18	(0.76 to 1.82)	.47
Surgery Approach												
Laparoscopic	1.00						1.00					
Open	0.98	(0.68 to 1.41)	.91				0.98	(0.67 to 1.43)	.92			
Gastrectomy												
Total	1.00			1.00			1.00					
Distal	0.64	(0.44 to 0.92)	.02	1.03	(0.69 to 1.55)	.87	0.58	(0.39 to 0.86)	.007	0.96	(0.61 to 1.51)	.86
Proximal	0.53	(0.21 to 1.33)	.18	1.10	(0.43 to 2.82)	.85	0.44	(0.16 to 1.21)	.11	0.88	(0.30 to 2.54)	.81
A-T	3.11	(1.13 to 8.57)	.03	1.62	(0.54 to 4.88)	.39	3.18	(1.15 to 8.77)	.03	1.95	(0.63 to 5.98)	.24
Complications, CD												
0	1.00						1.00					
1–2	0.85	(0.53 to 1.38)	.51				0.94	(0.57 to 1.53)	.79			
3–4	1.15	(0.68 to 1.94)	.60				1.22	(0.71 to 2.09)	.48			
Location												
Upper	1.00						1.00					
Middle	1.11	(0.65 to 1.92)	.70				0.94	(0.52 to 1.71)	.85			
Lower	0.99	(0.65 to 1.5)	.95				0.97	(0.63 to 1.49)	.88			
Diffuse	3.25	(1.56 to 6.80)	.002^a^				3.53	(1.68 to 7.41)	.001^a^			
Diameter (cm)												
≤2	1.00			1.00			1.00					
2–5	1.49	(1.00 to 2.23)	.05	1.20	(0.79 to 1.82)	.40	1.52	(0.99 to 2.33)	.05	1.22	(0.79 to 1.90)	.38
>5	2.61	(1.60 to 4.25)	<.001	1.23	(0.71 to 2.12)	.46	2.8	(1.69 to 4.64)	<.001	1.27	(0.72 to 2.25)	.41
Differentiation												
Well-Moderate	1.00						1.00					
Poor	1.40	(0.98 to 1.99)	.07				1.46	(1.00 to 2.12)	.05^b^	1.04	(0.69 to 1.56)	.85
Signet Ring												
No	1.00						1.00					
Yes	1.43	(0.92 to 2.22)	.11				1.53	(0.97 to 2.41)	.06			
Vascular invasion												
No	1.00			1.00			1.00					
Yes	2.72	(1.90 to 3.89)	<.001	0.99	(0.63 to 1.55)	.96	2.77	(1.90 to 4.03)	<.001	1.12	(0.71 to 1.77)	.63
ypT												
T0–1	1.00						1.00					
T2	3.42	(1.35 to 8.67)	.01	2.34	(0.78 to 7.07)	.13	3.14	(1.23 to 8.03)	.02	2.02	(0.66 to 6.14)	.22
T3	3.91	(1.60 to 9.54)	.003	2.34	(0.79 to 6.89)	.12	3.69	(1.51 to 9.02)	.004	1.96	(0.66 to 5.84)	.23
T4	8.51	(3.70 to 19.56)	<.001	3.82	(1.34 to 10.92)	.01	7.06	(3.06 to 16.29)	<.001	2.91	(1.01 to 8.41)	.05^b^
ypN												
N0	1.00			1.00			1.00					
N1	1.75	(0.98 to 3.13)	.06	1.33	(0.71 to 2.48)	.38	1.76	(0.95 to 3.24)	.07	1.36	(0.69 to 2.71)	.38
N2	3.94	(2.33 to 6.67)	<.001	2.51	(1.42 to 4.41)	.001	3.68	(2.10 to 6.45)	<.001	2.28	(1.22 to 4.27)	.01
N3	8.73	(5.39 to 14.15)	<.001	5.35	(3.11 to 9.22)	<.001	8.69	(5.25 to 14.4)	<.001	4.95	(2.56 to 9.57)	<.001
pCR												
No	1.00			1.00								
Yes	0.18	(0.04 to 0.73)	.02	0.99	(0.18 to 5.52)	.99	0.20	(0.05 to 0.83)	.03	1.03	(0.18 to 5.75)	.98
NACT												
≤2	1.00						1.00					
>2	0.87	(0.60 to 1.25)	.45				0.83	(0.57 to 1.22)	.34			
NACT Regime												
SOX	1.00						1.00					
XELOX	0.86	(0.58 to 1.29)	.47				0.85	(0.56 to 1.30)	.46			
AC cycle												
≤4	1.00						1.00					
>4	1.02	(0.72 to 1.46)	.90				0.98	(0.67 to 1.42)	.90			
AC Regime												
SOX or other^c^	1.00											
XELOX	0.67	(0.43 to 1.03)	.07				0.65	(0.41 to 1.02)	.06			
PECTI, weeks												
≤9	1.00			1.00			1.00			1.00		
9–13	1.75	(1.10 to 2.78)	.02	1.53	(0.95 to 2.47)	.08	1.71	(1.05 to 2.78)	.03	1.46	(0.88 to 2.41)	.14
>13	2.22	(1.29 to 3.84)	.004	1.98	(1.12 to 3.49)	.02	2.26	(1.28 to 3.99)	.005	1.94	(1.06 to 3.53)	.03
TTS, weeks												
≤5	1.00						1.00					
>5	1.56	(1.09 to 2.23)	.01^d^				1.51	(1.04 to 2.19)	.03^d^			
TAC, weeks												
≤6	1.00						1.00					
>6	1.17	(0.81 to 1.69)	.40				1.18	(0.80 to 1.74)	.40			

Values in parentheses are 95% confidence intervals; 'aIt was not included into multivariate cox analysis due to subgroup volume limitation; ^b^The non-approximate P-value <0.05; ^C^Other regimes included S-1 (n = 13) and S-1 or capecitabine plus paclitaxel (n = 12); ^d^ They were not included into multivariate Cox analysis due to severe multicollinearity problem.

HR, hazard ratio; BMI, body mass index; ASA, American Society of Anesthesiologists; ECOG, Eastern Cooperative Oncology Group; AT, abdominal-thoracic surgery; CD, Clavein–Dindo Classification; pCR, pathological complete response; NACT, neoadjuvant chemotherapy; SOX, S-1 plus oxaliplatin chemotherapy; XELOX, capecitabine plus oxaliplatin chemotherapy; AC, adjuvant chemotherapy; PECTI, perioperative chemotherapy time interval; TTS, time to surgery; TAC, time to adjuvant chemotherapy.

**Figure 5:**
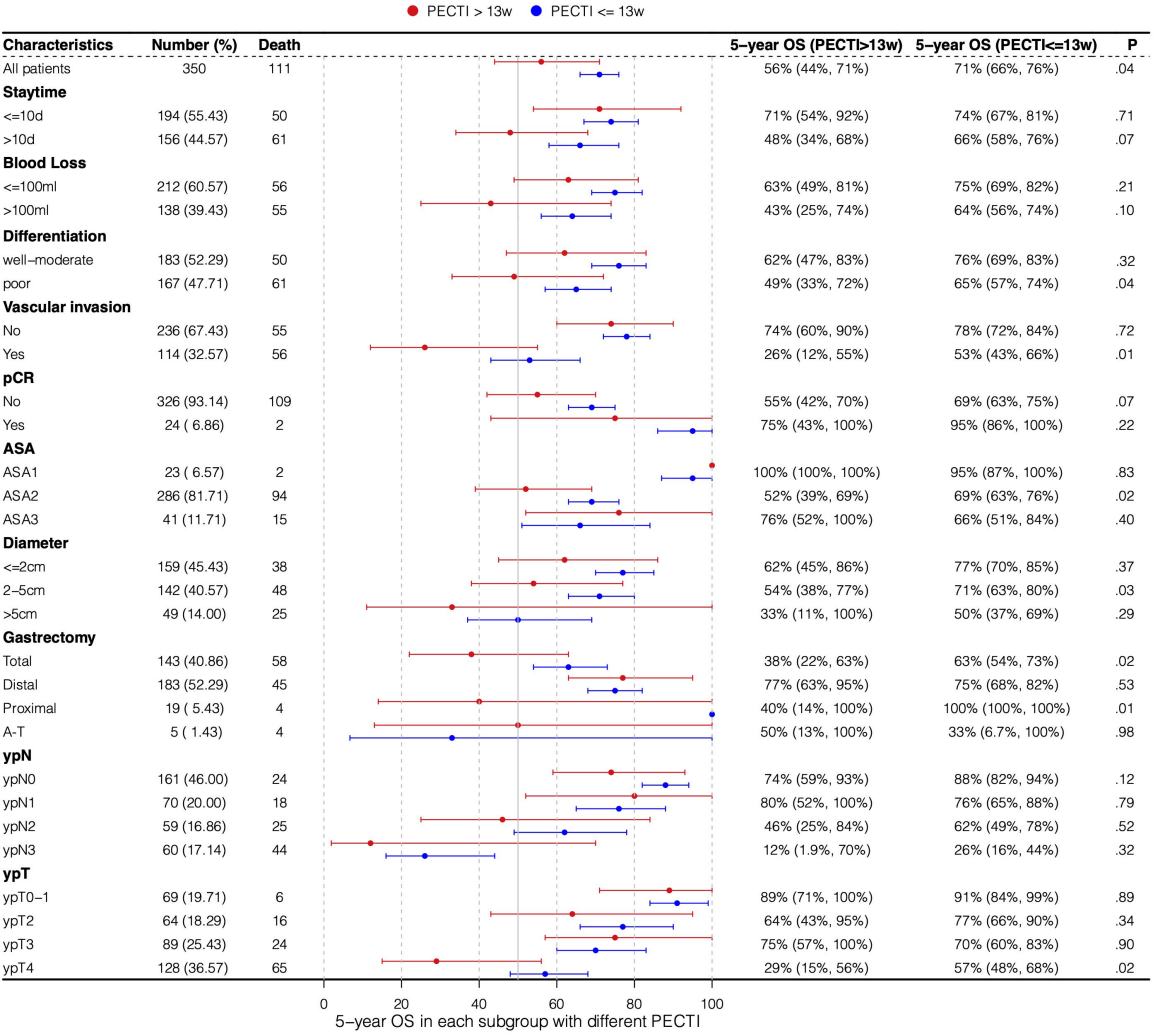
Subgroup analyses by forest plot based on 5-year survival rate. Plot showing the 5-year survival rate of the population experiencing PECTI within or over 13 weeks. PECTI, perioperative chemotherapy time interval; OS, overall survival; pCR, pathological complete response; ASA, American Society of Anesthesiologists; AT, abdominal-thoracic surgery.

A forest plot of subgroup analyses based on survival rate was drawn to show their influence on 5-year survival outcomes in patients, as shown in **Figure 5**. OS at 5 years was 71% (95% CI, 66% to 76%) with PECTI within 13 weeks but 56% (95% CI, 44% to 71%) with PECTI over 13 weeks (P = .04). Furthermore, in subgroup analyses, compared within 13 weeks, patients with PECTI over 13 weeks presented a significant decrease in OS in the case of poor differentiation (P = .04), and positive vascular invasion (P = .01), and ASA2 (P = .02), and diameter of 2–5 cm (P = .03), and total gastrectomy (P = .02), and ypT4 (P = .02).

### 3.3 Prognostic nomogram

**Figure 6:**
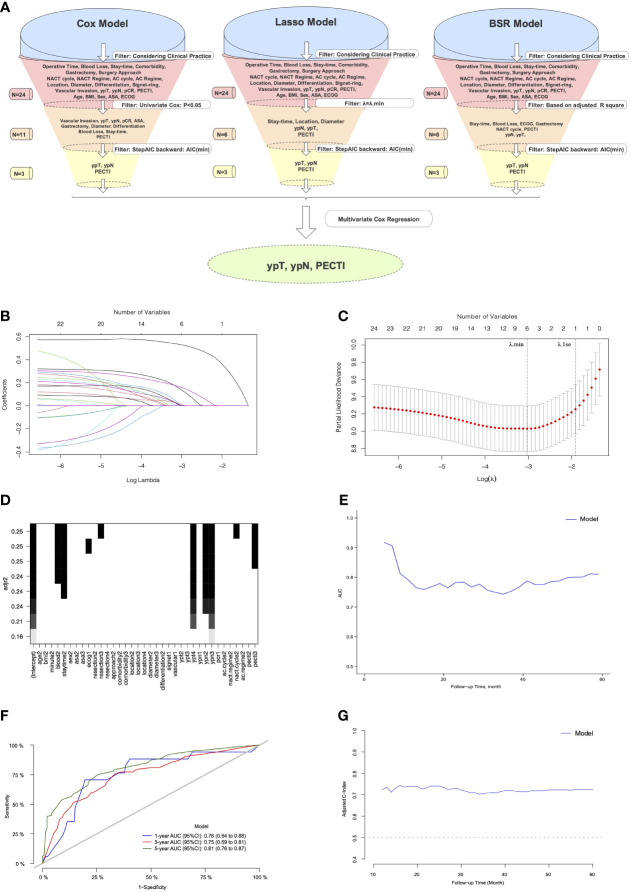
The process of variables into nomogram by filtration and discrimination of model. **(A)** The Illustration of Process. The variables were selected by comparison among the Cox, Lasso, and BSR models. Considering clinical practice, a total of 24 variables were eligible into models’ filtration, respectively. First filter was different: variables from the Cox model was filtered through univariate Cox analyses (P <.05), and variables from the Lasso model was filtered through penalty parameter selection (λ = λ.min), and variables from BSR model was filtered through adjusted R square. The remaining variables through the first filter would be continued to be filtered through minimum backward stepwise AIC by second filter and multivariate Cox regression by third filter, respectively. The model consisting of eventually remaining variables would be chosen for development of prognostic Nomogram model. **(B)** The LASSO coefficient profiles of the 24 variables; **(C)** Tuning parameter (λ) selection in the LASSO model. Dotted vertical lines were drawn at the optimal values using the minimum criteria (λ.min) and the 1 standard error of the minimum criteria (λ.1se); **(D)** Variable selection in BSR by adjusted R square. **(E)** The Model’s AUC with follow-up time. **(F)** The Model’s AUC in 1,3,5 years. **(G)** The Model’s adjusted C-index with follow-up time. Lasso, the least absolute shrinkage and selection operator; BSR, best subgroup regression; NACT, neoadjuvant chemotherapy; AC, adjuvant chemotherapy; pCR, pathological complete response; PECTI, perioperative chemotherapy time interval; BMI, body mass index; ASA, American Society of Anesthesiologists; ECOG, Eastern Cooperative Oncology Group; AUC, area under the curve; AIC, Akaike information criterion.

**Figure 7:**
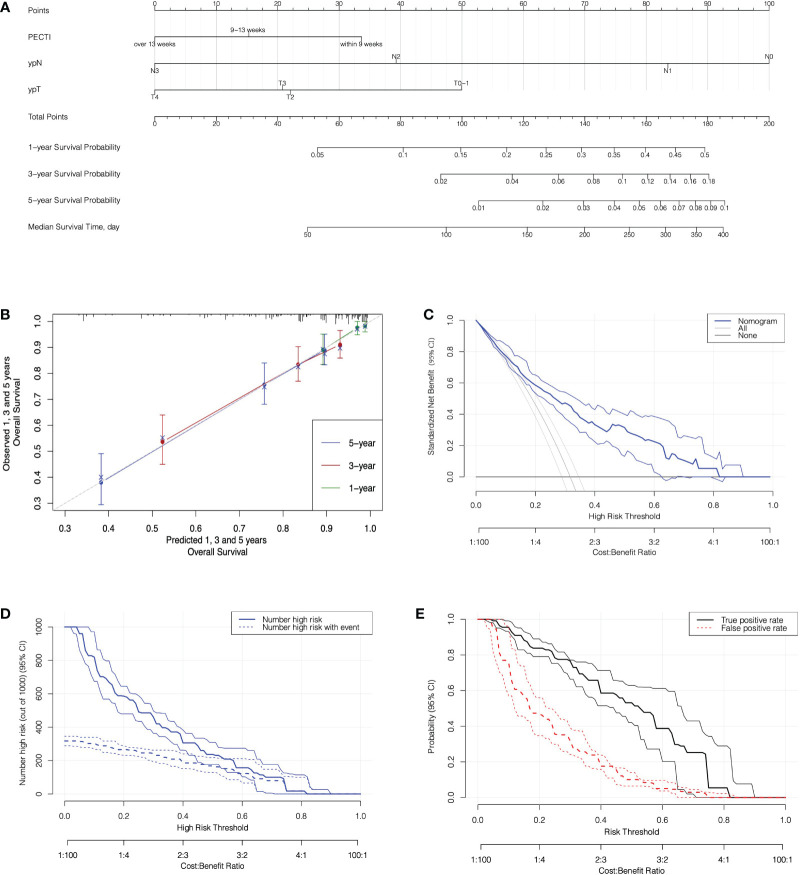
The nomogram prediction model with calibration and decision analyses. **(A)** The Nomogram Prediction Model. Points were assigned for PECTI, ypN, and ypT by drawing a line upward from the relevant values to the points line. The sum of the three points plotted on the total points line corresponds to assessments of 1-, 3-, and 5-year overall survival probability and median survival time of patients experiencing perioperative chemotherapy and surgery. **(B)** The Calibration of Nomogram. The dotted gray line expressed desired nomogram accuracy, and green, red, blue line expressed developed nomogram respectively in 1, 3, and 5 years, closer alignment with the gray line and better prognostic prediction. Crosses mark expressed bias corrected estimates and vertical bars expressed 95% Confidence Interval. **(C)** Decision curves for Nomogram model. Solid transverse line ‘None’ meant net benefit when all patients were still alive and diagonal straight line in left meant net benefit when all patients died. **(D)** Clinical impact curve for nomogram. Of 1,000 patients, the solid line shows the total number who would be deemed high risk of recurrence or death for each risk threshold, and the dashed line shows how many of those would be true. **(E)** True and false positive rates as functions of the risk threshold. Bands on all plots denote pointwise 95% CI constructed *via* bootstrapping. The horizontal axis is labeled in terms of both risk threshold and cost/benefit ratio, prompting the correspondence of them. PECTI, perioperative chemotherapy time interval; DCA, decision curve analyses.

The variable selection process is shown in **Figure 6A**, which illustrate that variables were first screened considering clinical practice, and then 24 variables were approved into three models (the Cox, Lasso, and BSR models). **Figures 6B, C** show the variable filtration process in the Lasso Model, and **Figure 6D** shows the variable filtration in the BSR Model. Comparisons were performed among the three models, and eventually, their filtration results were consistent with a 5-year AUC (0.81, 95% CI −0.76 to 0.87) and minimum AIC (1,158.91), which included the variables PECTI, ypT, and ypN. Model discrimination analyses showed an AUC of 0.81 (95% CI −0.76 to 0.87) at 5 years (**Figures 6E, F**). The model’s adjusted C-index was 0.73, and variations with follow-up time are shown in **Figure 6G**. Based on the results, the 1-, 3-, and 5-year survival probability and median survival time could be estimated by the nomogram described in **Figure 7A**. The calibration plot (**Figure 7B**) revealed good model consistency. N ∗ K cross-validation was performed for the nomogram model, and the mean AUC was 0.79 (IQR, 0.75 to 0.83) at 5 years when N = 400 and N = 5 (**Table S3**). DCA and CIC indicated that patients could obtain better clinical benefits using the developed nomogram, which had a relatively high true-positive rate under a wide range of risk thresholds (**Figures 7C–E**).

## 4 Discussion

In this study, we further investigated the time intervals between multimodal therapies influencing gastric cancer patients’ survival outcomes and then developed a nomogram model to predict survival, which can guide us to make clinical decisions regarding interventions and follow-up strategies. To our knowledge, our study is currently the first to systematically formulate the time interval concerning PECTI, TTS, and TAC correlation and their significance to gastric cancer patients’ survival outcomes and to develop a prognostic nomogram model for clinical reference.

Our results showed that the extended time interval from the completion of neoadjuvant chemotherapy to the initiation of adjuvant chemotherapy was unbeneficial for gastric cancer patients receiving perioperative chemotherapy and surgery. Patients would get a significantly better survival outcome both OS and PFS when PECTI within 9 weeks, and that would get worse with time interval extension so that patients would significantly decrease 23% in 5 years OS and 22% in 5 years PFS as PECTI over 13 weeks. In stratum analyses of the survival curve, extended PECTI may have worse survival on patients with poor differentiation, vascular invasion, total gastrectomy and ypT4, which meant these patients should receive the multimodality management with shorter PECTI. In addition, it was confirmed that the time to surgery within 5 weeks after accomplishment of NACT would have a significantly better OS and PFS than LAGC patients in the PECP population, which was consistent with our previous study conclusion for patients in the NACP population (9). Furthermore, it was also confirmed that time to chemotherapy within 6 weeks would not have an correlation with either OS or PFS of patients in the PECP population, consistent with our previous study for patients in the POCP population (10). It was peculiar and watchable that TTS out of range was negative for patient survival, but TAC was not. There were some explanations for the intriguing phenomena: (1) NACT could reduce tumor loading, degrade the staging, and afford operation possibility, but the extension to surgery after NACT may give the tumor more time to renew and mutate to a more tenacious solid with intricate immune microenvironment changes. Thus, surgery within a specific time window to strangle residual neoplasms was imperative and indispensable. (2) Shorter or extended TAC was of no help to the matter both OS and PFS in population of PECP because NACT and surgery had removed almost all of the tumor so that a few cancer cells would not grow exponentially due to small cardinal number. Hence, patients in the PECP population were more tolerant of delayed initiation of AC, and it was beneficial for patients to recover from surgery mentally, physically and economically. In relationship and linearity analyses, the PECTI effect was thought to be mediated mainly by TTS. However, we could not deny the role of TAC because it was also correlated with survival outcomes independently in the only POCP population in our previous study (10). Given the complicated interrelation of TTS and TAC, PECTI (TTS plus TAC) was more comprehensive and flexible. The growth of gastric cancer cells was time-consuming in the range of perioperative period, and the control of PECTI was more reasonable. Just in control of PECTI, TTS could be extended appropriately and shortened TAC could make a remedy, and delayed TAC might be allowed when TTS was shortened. Therefore, considering the extra separate multivariate Cox analyses (**Table S2**) and discussion above, PECTI as a whole is better than any part of them to predict survival outcomes.

To construct an efficient clinical prognostic model, we devised a standard variable filtration mechanism. The Cox, Lasso, and BSR models similarly singled out three variables (PECTI, ypN, and ypN), meaning that they were indispensable for prognosis. The developed model obtained a 5-year minimum AIC, maximum AUC and C-index in our cohort and an obvious net benefit in decision curve analyses. Therefore, it was valuable for clinical use after verification of calibration, discrimination and DCA. However, some points should be considered: (1) Interventions are recommended by increasing AC cycles, dosage, and follow-up frequency for patients with a high risk of recurrence or death predicted by a prognostic model when AC is scheduled after surgery. (2) The concept of net benefit displayed in DCA is applicable to the assessment of intervention policies but not to individuals (16).

However, there have been some paradoxical findings in many cancers. For instance, breast cancer was reported that TTS within 8 (17), 6 (18), or 3 (19) weeks was more beneficial to operable breast cancer patients, contrary to studies showing that TTS was insignificant (20, 21). One reason was that different tumors with diverse biological characteristics treated with various chemotherapy are expected to show different dynamics. Regarding gastric cancer, Liu et al. reported that TTS was not crucial to long-term survival outcomes, although TTS presented higher odds of pCR when TTS was over 6 weeks in the NACP plus PECP population (7). Brenkman et al. reported that TAC did not significantly influence OS within 6 weeks, 6–8 weeks, and over 8 weeks in the PECP population (8), which was consistent with our results. However, in contrast, Juan et al. reported that in a study of 60 patients, TTS was not associated with pCR, downstaging, or OS among TTS within 4 weeks, 4–6 weeks, and over 6 weeks (22). Overall, the disparities among studies of the same cancer may be attributed to the following reasons: (1) retrospective sample limitations; (2) investigated population discrepancies among PECP, NACP, and POCP; and (3) diverse chemotherapy regimens such as SOX/XELOX or FOLFOX (folinic acid, fluorouracil, and oxaliplatin). (4) The TI subgroup division method is vital, and significance might be concealed at some chosen cutoff points. Concerning PECTI, three related studies recently confirmed its value in ovarian cancer. Patients with shorter PECTI (within 6 weeks) had better OS and PFS (23), and then Searle et al. confirmed a benefit in OS as PECTI within 10 weeks (24), and Wang et al. confirmed a benefit in both OS and PFS outcomes as PECTI within 5 weeks (25). There are some possible explanations for the correlation of longer PECTI with worse OS or PFS. (1) Unlike some cancers acceptable with delaying time intervals, some cancer cells, such as gastric cancer, are highly heterogeneous and malignant, so the postponement of treatment may give cells time to renew and evolve. Therefore, a longer PECTI might provide a time window for gastric cancer cells to grow. (2) We still found that PECTI longer than 13 weeks had higher ECOG assessment with an approaching significance (P = .05) compared to PECTI shorter than 9 weeks, which might indicate that fitter patients received therapy more quickly and then had better survival outcomes. Generally, in the majority of cancer studies, longer TTS or PECTI would present worse long-term survival outcomes either in OS or PFS.

Nevertheless, our study had several limitations, as follows: (1) The study was a retrospective study performed in a single center, and censoring in cohorts may induce study bias. (2) Lack of details: why the patients’ treatment schedule was postponed was unclear, and bias might arise. (3) Some potential variables might not be included and remain to be excavated, and external validation of the model was absent. (4) The variables “age” and “stay-time” were significantly different between PECTI groups in the patients’ characteristics. However, through statistical calculations by our statistician, they were negligible and could be ignored. Moreover, a univariate Cox analysis was performed and it indicated that the variable “age” would not affect other clinical factors or prognosis. Lastly, the gap was not obvious that the median age was 59, 59, and 63 years, respectively, and the median stay-time was 10, 10, and 12 days, respectively, among different PECTI groups.

In summary, for locally advanced and operable gastric patients receiving perioperative SOX/XELOX chemotherapy, the shorter time interval from accomplishment of NACT to initiation of AC might be recommended for its significantly positive correlation with patient PFS and OS, and this effect might be mediated mainly by TTS. Prognostic nomograms (including PECTI, ypT, and ypN) could be used clinically by patients and doctors to predict long-term survival probabilities and help guide clinical interventions or postoperative schedules. Even under COVID-19 circumstances, the time interval between perioperative chemotherapy and surgery should still be shortened to the appropriate time frame as much as possible. Prospective studies and multicenter studies are needed to confirm our conclusion, and more data are needed to improve our nomogram model.

## Data availability statement

The original contributions presented in the study are included in the article/**Supplementary Material**. Further inquiries can be directed to the corresponding authors.

## Ethics statement

The studies involving human participants were reviewed and approved by the Peking University Cancer Hospital Ethics Committee. The patients/participants provided their written informed consent to participate in this study.

## Author contributions

Z-YL and FS contributed to the collection of funding. All authors contributed to the article and approved the submitted version.
