# Supplementary material for: The significance of time interval between perioperative SOX/XELOX chemotherapy and clinical decision model in gastric cancer
Source: Front Oncol. 2022 Dec 23;12:956706. doi: 10.3389/fonc.2022.956706 (PMC9816861; doi:10.3389/fonc.2022.956706)
Supplement: Supplementary file 1 [file Image_1.pdf]

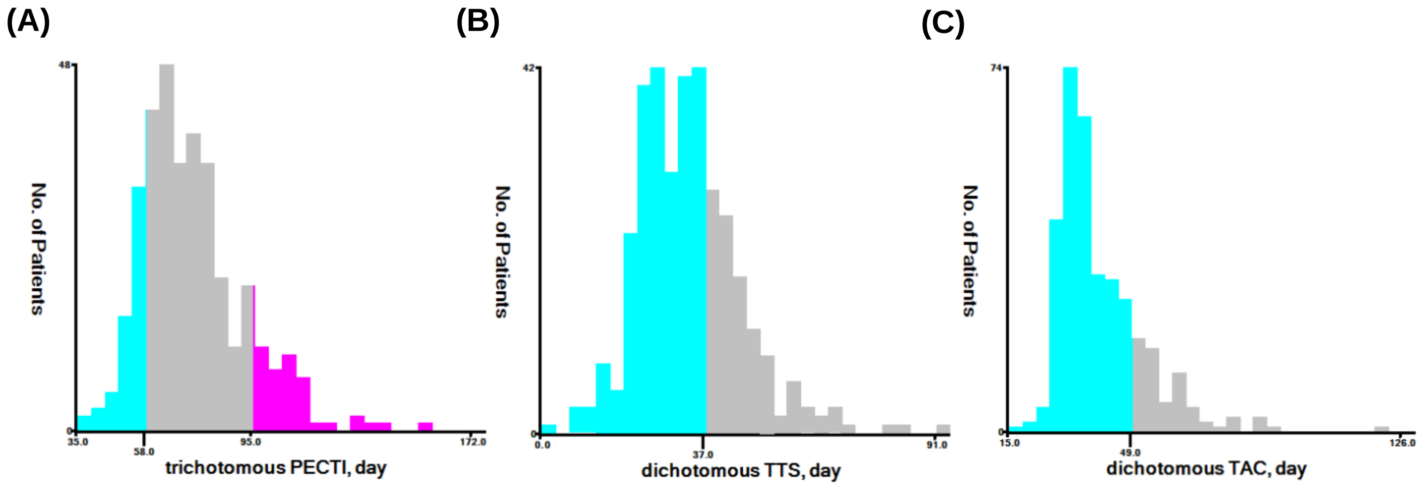

Supplementary Figure 1. The Patients Cutoff-finding Histograms Generated by X-tile Tool. (A) PECTI group. (B)TTS group. (C) TAC group. The X-tile cutoff calculation was based on Kaplan-Meier methods. Abbreviation: PECTI: Perioperative Chemotherapy Time Interval; TTS: Time to Surgery; TAC: Time to Adjuvant Chemotherapy.
