# Supplementary material for: The significance of time interval between perioperative SOX/XELOX chemotherapy and clinical decision model in gastric cancer
Source: Front Oncol. 2022 Dec 23;12:956706. doi: 10.3389/fonc.2022.956706 (PMC9816861; doi:10.3389/fonc.2022.956706)
Supplement: Supplementary file 2 [file Image_2.pdf]

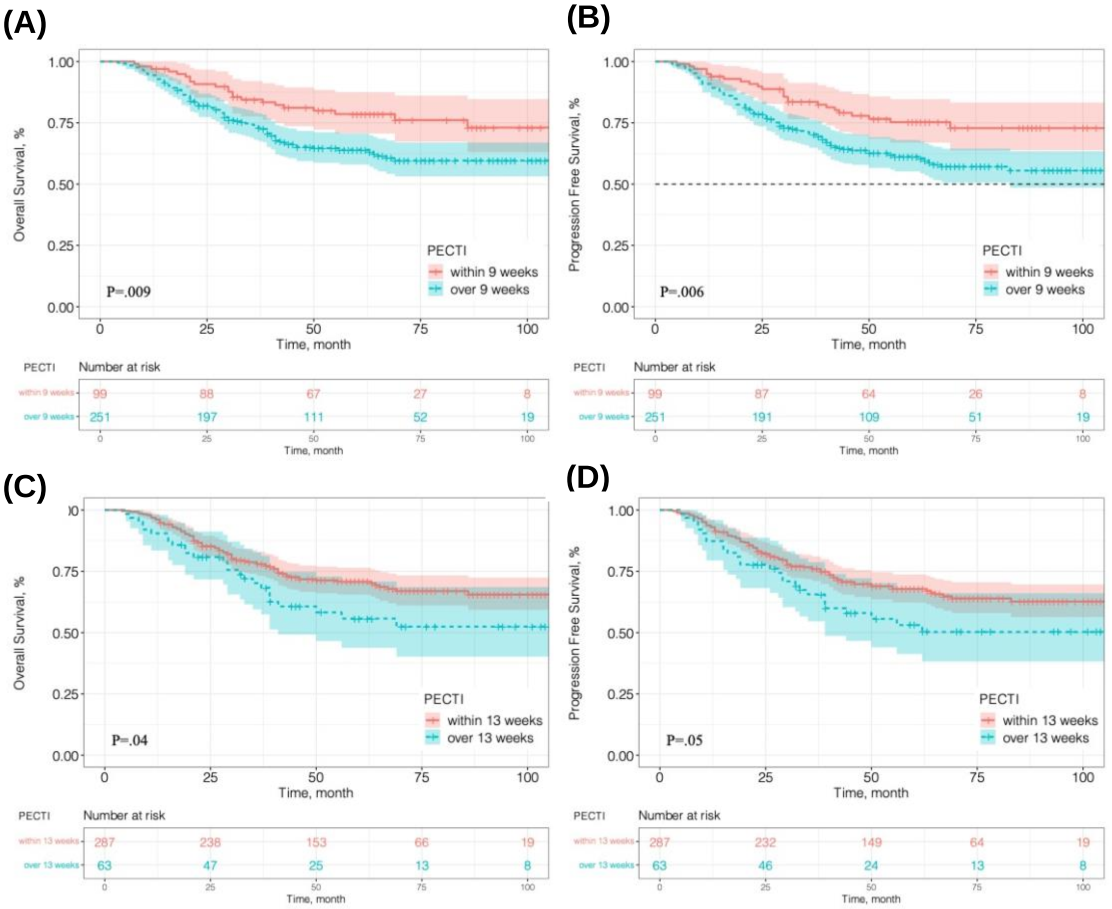

Supplementary Figure 2. The Kaplan-Meier Survival Analyses in Dichotomous PECTI Group. (A) OS in dichotomous PECTI study group with cutoff of 9 weeks; (B) PFS in dichotomous PECTI study group with cutoff of 9 weeks; (C) OS in dichotomous PECTI study group with cutoff of 13 weeks; (D) PFS in dichotomous PECTI study group with cutoff of 13-weeks. Abbreviation: PECTI: Perioperative Chemotherapy Time Interval.
