# Supplementary material for: The significance of time interval between perioperative SOX/XELOX chemotherapy and clinical decision model in gastric cancer
Source: Front Oncol. 2022 Dec 23;12:956706. doi: 10.3389/fonc.2022.956706 (PMC9816861; doi:10.3389/fonc.2022.956706)
Supplement: Supplementary file 3 [file Table_1.docx]

**Table S1. Patients Demography and Clinicopathologic Characteristics in TTS and TAC Groups**

| **Characteristics ^a^** |  | **All patients** | **TTS, weeks** | | | **TAC, weeks** | | |
| --- | --- | --- | --- | --- | --- | --- | --- | --- |
|  |  |  | **≤ 5 weeks** | **> 5 weeks** | **p** | **≤ 6 weeks** | **> 6 weeks** | **p** |
| Patients Number |  | 350 | 209/350 (59.71) | 141/350 (40.29) |  | 232/350 (66.29) | 118/350 (33.71) |  |
| Age, median [IQR], y |  | 60.00 [53.00 to 64.00] | 59.00 [53.00 to 64.00] | 60.00 [53.00 to 66.00] | .24 | 58.00 [52.00 to 63.00] | 61.00 [56.25 to 66.00] | .001 |
| BMI, median [IQR], (kg/m2) |  | 23.74 [21.63 to 26.00] | 23.73 [21.71 to 25.95] | 23.81 [21.45 to 26.15] | .85 | 23.88 [21.79 to 26.28] | 23.28 [21.11 to 25.50] | .06 |
| Operative Time [IQR], min |  | 202.50 [171.00 to 243.75] | 201.00 [169.00 to 244.00] | 204.00 [175.00 to 242.00] | .80 | 205.50 [170.75 to 244.00] | 200.00 [171.25 to 242.00] | .85 |
| Blood Loss [IQR], ml |  | 100.00 [80.00 to 179.25] | 100.00 [80.00 to 150.00] | 100.00 [85.00 to 200.00] | .69 | 100.00 [78.75 to 186.00] | 100.00 [100.00 to 159.75] | .47 |
| Stay-time [IQR], days |  | 10.00 [9.00 to 13.00] | 10.00 [9.00 to 13.00] | 10.00 [9.00 to 14.00] | .49 | 10.00 [9.00 to 12.00] | 12.00 [9.00 to 15.75] | <0.001 |
| Sex (%) | Male | 273/350 (78.00) | 158/209 (75.60) | 115/141 (81.56) | .23 | 182/232 (78.45) | 91/118 (77.12) | .88 |
|  | Female | 77/350 (22.00) | 51/209 (24.40) | 26/141 (18.44) |  | 50/232 (21.55) | 27/118 (22.88) |  |
| ASA (%) | 1 | 23/350 (6.57) | 16/209 (7.66) | 7/141 (4.96) | .59 | 16/232 (6.90) | 7/118 (5.93) | .89 |
|  | 2 | 286/350 (81.71) | 168/209 (80.38) | 118/141 (83.69) |  | 188/232 (81.03) | 98/118 (83.05) |  |
|  | 3 | 41/350 (11.71) | 25/209 (11.96) | 16/141 (11.35) |  | 28/232 (12.07) | 13/118 (11.02) |  |
| ECOG (%) | 0 | 264/350 (75.43) | 162/209 (77.51) | 102/141 (72.34) | .33 | 179/232 (77.16) | 85/118 (72.03) | .36 |
|  | 1~3 | 86/350 (24.57) | 47/209 (22.49) | 39/141 (27.66) |  | 53/232 (22.84) | 33/118 (27.97) |  |
| Gastrectomy (%) ^b^ | Total | 143/350 (40.86) | 85/209 (40.67) | 58/141 (41.13) | .70 | 90/232 (38.79) | 53/118 (44.92) | .23 |
|  | Distal | 183/350 (52.29) | 109/209 (52.15) | 74/141 (52.48) |  | 129/232 (55.60) | 54/118 (45.76) |  |
|  | Proximal | 19/350 (5.43) | 13/209 (6.22) | 6/141 (4.26) |  | 11/232 (4.74) | 8/118 (6.78) |  |
|  | A-T | 5/350 (1.43) | 2/209 (0.96) | 3/141 (2.13) |  | 2/232 (0.86) | 3/118 (2.54) |  |
| Surgery Approach (%) | Laparoscopic | 163/350 (46.57) | 96/209 (45.93) | 67/141 (47.52) | .86 | 104/232 (44.83) | 59/118 (50.00) | .42 |
|  | Open | 187/350 (53.43) | 113/209 (54.07) | 74/141 (52.48) |  | 128/232 (55.17) | 59/118 (50.00) |  |
| Complications, CD (%) | 0 | 244/350 (69.71) | 146/209 (69.86) | 98/141 (69.50) | .77 | 173/232 (74.57) | 71/118 (60.17) | .01 |
|  | 1-2 | 66/350 (18.86) | 41/209 (19.62) | 25/141 (17.73) |  | 39/232 (16.81) | 27/118 (22.88) |  |
|  | 3-4 | 40/350 (11.43) | 22/209 (10.53) | 18/141 (12.77) |  | 20/232 (8.62) | 20/118 (16.95) |  |
| Location (%) | Upper | 105/350 (30.00) | 64/209 (30.62) | 41/141 (29.08) | .58 | 62/232 (26.72) | 43/118 (36.44) | .29 |
|  | Middle | 54/350 (15.43) | 31/209 (14.83) | 23/141 (16.31) |  | 36/232 (15.52) | 18/118 (15.25) |  |
|  | Lower | 177/350 (50.57) | 108/209 (51.67) | 69/141 (48.94) |  | 124/232 (53.45) | 53/118 (44.92) |  |
|  | Diffuse | 14/350 (4.00) | 6/209 (2.87) | 8/141 (5.67) |  | 10/232 (4.31) | 4/118 (3.39) |  |
| Diameter (%) | ≤2 | 159/350 (45.43) | 96/209 (45.93) | 63/141 (44.68) | .92 | 103/232 (44.40) | 56/118 (47.46) | .34 |
|  | 2-5 | 142/350 (40.57) | 85/209 (40.67) | 57/141 (40.43) |  | 92/232 (39.66) | 50/118 (42.37) |  |
|  | >5 | 49/350 (14.00) | 28/209 (13.40) | 21/141 (14.89) |  | 37/232 (15.95) | 12/118 (10.17) |  |
| Differentiation (%) | Well-Moderate | 183/350 (52.29) | 111/209 (53.11) | 72/141 (51.06) | .79 | 121/232 (52.16) | 62/118 (52.54) | .99 |
|  | Poor | 167/350 (47.71) | 98/209 (46.89) | 69/141 (48.94) |  | 111/232 (47.84) | 56/118 (47.46) |  |
| Signet-ring (%) | No | 292/350 (83.43) | 182/209 (87.08) | 110/141 (78.01) | .04 | 195/232 (84.05) | 97/118 (82.20) | .77 |
|  | Yes | 58/350 (16.57) | 27/209 (12.92) | 31/141 (21.99) |  | 37/232 (15.95) | 21/118 (17.80) |  |
| Vascular Invasion (%) | No | 236/350 (67.43) | 150/209 (71.77) | 86/141 (60.99) | .05 | 159/232 (68.53) | 77/118 (65.25) | .62 |
|  | Yes | 114/350 (32.57) | 59/209 (28.23) | 55/141 (39.01) |  | 73/232 (31.47) | 41/118 (34.75) |  |
| ypT (%) | T0 | 27/350 (7.71) | 15/209 (7.18) | 12/141 (8.51) | .90 | 18/232 (7.76) | 9/118 (7.63) | .98 |
|  | T1 | 42/350 (12.00) | 24/209 (11.48) | 18/141 (12.77) |  | 29/232 (12.50) | 13/118 (11.02) |  |
|  | T2 | 64/350 (18.29) | 40/209 (19.14) | 24/141 (17.02) |  | 42/232 (18.10) | 22/118 (18.64) |  |
|  | T3 | 89/350 (25.43) | 56/209 (26.79) | 33/141 (23.40) |  | 57/232 (24.57) | 32/118 (27.12) |  |
|  | T4 | 128/350 (36.57) | 74/209 (35.41) | 54/141 (38.30) |  | 86/232 (37.07) | 42/118 (35.59) |  |
| ypN (%) | N0 | 161/350 (46.00) | 98/209 (46.89) | 63/141 (44.68) | .25 | 105/232 (45.26) | 56/118 (47.46) | .43 |
|  | N1 | 70/350 (20.00) | 45/209 (21.53) | 25/141 (17.73) |  | 52/232 (22.41) | 18/118 (15.25) |  |
|  | N2 | 59/350 (16.86) | 37/209 (17.70) | 22/141 (15.60) |  | 38/232 (16.38) | 21/118 (17.80) |  |
|  | N3 | 60/350 (17.14) | 29/209 (13.88) | 31/141 (21.99) |  | 37/232 (15.95) | 23/118 (19.49) |  |
| pCR (%) | No | 326/350 (93.14) | 197/209 (94.26) | 129/141 (91.49) | .43 | 217/232 (93.53) | 109/118 (92.37) | .86 |
|  | Yes | 24/350 (6.86) | 12/209 (5.74) | 12/141 (8.51) |  | 15/232 (6.47) | 9/118 (7.63) |  |
| NACT Cycle (%) | ≤2 | 181/350 (51.71) | 107/209 (51.20) | 74/141 (52.48) | .90 | 119/232 (51.29) | 62/118 (52.54) | .91 |
|  | >2 | 169/350 (48.29) | 102/209 (48.80) | 67/141 (47.52) |  | 113/232 (48.71) | 56/118 (47.46) |  |
| NACT Regime (%) | SOX | 238/350 (68.00) | 146/209 (69.86) | 92/141 (65.25) | .43 | 159/232 (68.53) | 79/118 (66.95) | .86 |
|  | XELOX | 112/350 (32.00) | 63/209 (30.14) | 49/141 (34.75) |  | 73/232 (31.47) | 39/118 (33.05) |  |
| AC Cycle (%) | ≤4 | 196/350 (56.00) | 119/209 (56.94) | 77/141 (54.61) | .75 | 121/232 (52.16) | 75/118 (63.56) | .06 |
|  | >4 | 154/350 (44.00) | 90/209 (43.06) | 64/141 (45.39) |  | 111/232 (47.84) | 43/118 (36.44) |  |
| AC Regime (%) | SOX or other ^c^ | 247/350 (70.57) | 149/209 (71.29) | 98/141 (69.50) | .81 | 164/232 (70.69) | 83/118 (70.34) | .99 |
|  | XELOX | 103/350 (29.43) | 60/209 (28.71) | 43/141 (30.50) |  | 68/232 (29.31) | 35/118 (29.66) |  |

^a^ Values in parentheses and brackets are percentages and interquartile range respectively. ^b^ Calculated by Fisher exact test. ^C^ Other regimes included S-1 (n=13) and S-1 or capecitabine plus paclitaxel (n=12).

Abbreviation: TTS: Time to Surgery; TAC: Time to Adjuvant Chemotherapy; IQR: Interquartile Range; BMI: Body Mass Index; ASA: American Society of Anesthesiologists; ECOG: Eastern Cooperative Oncology Group; AT: Abdominal-Thoracic surgery; CD:Clavein-Dindo Classification; pCR: pathological Complete Response; NACT: Neoadjuvant Chemotherapy; SOX: S-1 plus Oxaliplatin Chemotherapy; XELOX: Capecitabine plus Oxaliplatin Chemotherapy; AC: Adjuvant Chemotherapy.
