# Supplementary material for: The significance of time interval between perioperative SOX/XELOX chemotherapy and clinical decision model in gastric cancer
Source: Front Oncol. 2022 Dec 23;12:956706. doi: 10.3389/fonc.2022.956706 (PMC9816861; doi:10.3389/fonc.2022.956706)
Supplement: Supplementary file 4 [file Table_2.docx]

**Table S2. The Multivariate Survival Analyses Using Cox Proportional Hazards Model Between Time Interval Groups**

| **Characteristics** | **Progression Free Survival** | | | | | | **Overall Survival** | | | | | |
| --- | --- | --- | --- | --- | --- | --- | --- | --- | --- | --- | --- | --- |
|  | **Univariate HR (95% CI)** | | **P** | **Multivariate HR (95% CI)** | | **P** | **Univariate HR (95% CI)** | | **P** | **Multivariate HR (95% CI)** | | **P** |
| PECTI, weeks |  |  |  |  |  |  |  |  |  |  |  |  |
| ≤9 | 1.00 |  |  | 1.00 |  |  | 1.00 |  |  | 1.00 |  |  |
| 9-13 | 1.75 | (1.10 to 2.78) | .02 | 1.68 | (0.98, 2.86) | .06 | 1.71 | (1.05 to 2.78) | .03 | 1.68 | (0.97 to 2.94) | .07 |
| >13 | 2.22 | (1.29 to 3.84) | .004 | 2.14 | (0.99, 4.63) | .05 | 2.26 | (1.28 to 3.99) | .005 | 2.28 | (1.02 to 5.10) | .05^a^ |
| TTS, weeks |  |  |  |  |  |  |  |  |  |  |  |  |
| ≤5 | 1.00 |  |  | 1.00 |  |  | 1.00 |  |  | 1.00 |  |  |
| >5 | 1.56 | (1.09 to 2.23) | .01 | 1.18 | (0.76, 1.82) | .47 | 1.51 | (1.04 to 2.19) | .03 | 1.12 | (0.71 to 1.76) | .64 |
| TAC, weeks |  |  |  |  |  |  |  |  |  |  |  |  |
| ≤6 | 1.00 |  |  | 1.00 |  |  | 1.00 |  |  | 1.00 |  |  |
| >6 | 1.17 | (0.81 to 1.69) | .40 | 0.89 | (0.57, 1.40) | .61 | 1.18 | (0.80 to 1.74) | .40 | 0.88 | (0.55 to 1.41) | .59 |

Values in parentheses are 95% confidence intervals; ^a^ The non-approximate P value <0.05.

Abbreviation: HR: Hazard Ratio; PECTI: Perioperative Chemotherapy Time Interval; TTS: Time to Surgery; TAC: Time to Adjuvant Chemotherapy.
