# Supplementary material for: The significance of time interval between perioperative SOX/XELOX chemotherapy and clinical decision model in gastric cancer
Source: Front Oncol. 2022 Dec 23;12:956706. doi: 10.3389/fonc.2022.956706 (PMC9816861; doi:10.3389/fonc.2022.956706)
Supplement: Supplementary file 5 [file Table_3.docx]

**Table S3. N*K Cross Validation for Nomogram Model (N=400, K=5)**

| **OS** | **AUC** | **(95% CI)** | **mean AUC** | **(Interquartile Range)** |
| --- | --- | --- | --- | --- |
| 1-year | 0.75 | (0.63 to 0.87) | 0.74 | (0.66 to 0.85) |
| 2-year | 0.79 | (0.72 to 0.85) | 0.77 | (0.72 to 0.82) |
| 3-year | 0.75 | (0.69 to 0.82) | 0.73 | (0.69 to 0.78) |
| 4-year | 0.79 | (0.73 to 0.84) | 0.77 | (0.73 to 0.81) |
| 5-year | 0.81 | (0.76 to 0.87) | 0.79 | (0.75 to 0.83) |

The original sample would be divided into K subgroup including 4 training groups and 1 testing group and performing independent assortment for 5 times, and then iterated for 400 times so that there were 2000 AUC data generated and mean AUC would be figured out. Abbreviation: AUC: Area Under The Curve.
